# Engineering *Yarrowia lipolytica* for Campesterol Overproduction

**DOI:** 10.1371/journal.pone.0146773

**Published:** 2016-01-11

**Authors:** Hao-Xing Du, Wen-Hai Xiao, Ying Wang, Xiao Zhou, Yu Zhang, Duo Liu, Ying-Jin Yuan

**Affiliations:** 1 Key Laboratory of Systems Bioengineering (Ministry of Education), Tianjin University, Tianjin, China; 2 SynBio Research Platform, Collaborative Innovation Center of Chemical Science and Engineering (Tianjin), School of Chemical Engineering and Technology, Tianjin University, Tianjin, China; INRA, FRANCE

## Abstract

Campesterol is an important precursor for many sterol drugs, *e*.*g*. progesterone and hydrocortisone. In order to produce campesterol in *Yarrowia lipolytica*, C-22 desaturase encoding gene *ERG5* was disrupted and the heterologous 7-dehydrocholesterol reductase (DHCR7) encoding gene was constitutively expressed. The codon-optimized *DHCR7* from *Rallus norvegicus*, *Oryza saliva and Xenapus laevis* were explored and the strain with the gene *DHCR7* from *X*. *laevis* achieved the highest titer of campesterol due to D409 in substrate binding sites. In presence of glucose as the carbon source, higher biomass conversion yield and product yield were achieved in shake flask compared to that using glycerol and sunflower seed oil. Nevertheless, better cell growth rate was observed in medium with sunflower seed oil as the sole carbon source. Through high cell density fed-batch fermentation under carbon source restriction strategy, a titer of 453±24.7 mg/L campesterol was achieved with sunflower seed oil as the carbon source, which is the highest reported microbial titer known. Our study has greatly enhanced campesterol accumulation in *Y*. *lipolytica*, providing new insight into producing complex and desired molecules in microbes.

## Introduction

Campesterol is an important precursor for steroid drugs, which used as balance hormone drug in the pharmaceutical industry [1, 2]. Yeast is a conventional host for sterol production such as pregnenolone and hydrocortisone [3, 4], but campesterol tends to adhere to the membrane and forms membranes structure, which would be a burden to cells [5–7]. *Yarrowia lipolytica* lipid bodies accumulation is favorable for less polar compounds storage, thus *Y*. *lipolytica* is a promising host cell for hydrophobic metabolites production [8, 9]. Matthaus *et al*. found that lycopene located mainly within lipid bodies and increased lipid bodies formation led to an increase of lycopene storage capacity when lycopene was successfully produced in *Y*. *lipolytica* [10]. *Y*. *lipolytica* was also been used for the production of β-carotene [11], omega-3 fatty acids eicosapentaenoic acid (EPA) [12], citric acid [13–15] and microbial lipids for cocoa-butter substitute [15, 16] or biodiesel precursor [15, 17]. Thus, *Y*. *lipolytica* is a potential host to produce campesterol.

DHCR7 is a membrane-embedded enzyme, which reduces the seventh position of the carbon-carbon double bonds of ergosta-5,7-dienol to campesterol [3]. Zhou *et al*. once reported that DHCR7 activity was independent of cytochrome P450 reductase [18]. Some mutations in DHCR7 lead to human autosomal recessive metabolic disorder, known as the Smith-Lemli-Opitz syndrome (SLOS) [19]. Based on the crystal structure of its homological protein delta^14^-sterol reductase (MaSR1, PDB accession 4quv) from *Methylomicrobium alcaliphilum* 20Z, Li *et al*. [20] generated the structural model for human DHCR7 and found that majority of the SLOS-related mutations could be mapped to the sterol reductase catalytic domain, which comprised the carboxy-terminal half of the protein (the 6–10 helix) and affected the cofactor/sterol binding. However, there is no research which has clearly identified the specific sites in the substrate binding pocket for DHCR7 enzyme activity so far. Meanwhile, it is well known that screening enzymes from different species aided by computer docking is a conventional and efficient strategy to reveal the critical amino acid residue(s) in active pocket due to their enzyme structure diversity [21, 22]. Ding *et al*. [21] enhanced the taxadiene biosynthesis dramatically in yeast by selection of geranylgeranyl diphosphate synthase from six different species directed by computer-aided docking strategy. And it was also reported by Leonard *et al*. [22] that the optimization of geranylgeranyl diphosphate synthase and levopimaradiene synthase by phylogeny-based mutation strategy reduced the accumulation of byproducts significantly. Thus, DHCR7 from different species could be chosen and screened for a higher production of campesterol, aiding by protein modelling and cofactor/substrate docking for attempt to reveal the relationship between some specific sites and DHCR7 activity.

*Y*. *lipolytica* is capable to utilize many kinds of substrates such as glucose, glycerol and especially oil [23–25]. Manipulation of the employed carbon sources can tremendously vary the metabolic compounds synthesized in *Y*. *lipolytica*. When cultivated on hydrophilic carbon sources (*e*.*g*. glucose and glycerol), most of the *Y*. *lipolytica* strains do not store significant quantities of cellular lipids [13, 14, 17]. Meanwhile, growth of many *Y*. *lipolytica* strains on hydrophobic materials (*e*.*g*. fatty acids and triacylglycerols) is accompanied by remarkable accumulation of lipids [15, 16, 26], and the lipids accumulation is influenced by the fatty acid composition of the fat used as the substrate [15, 16, 26]. As lipids accumulation might be benefit for campesterol synthesis, exploration of different carbon sources would be one of the key points to enhance production.

Here, *Y*. *lipolytica* was selected as the host for campesterol production. Initially the DHCR7 was introduced into *Y*. *lipolytica* while bypass of ergosterol formation was disrupted. Screening of enzymes from different species and manipulation of different carbon source were utilized to enhance campesterol production. The strain with DHCR7 from *Xenapus laevis* grown in the medium containing sunflower seed oil achieved the highest campesterol production in 5-L bioreactor *via* high cell density fermentation process under carbon restriction strategy. This work provides a good reference to produce desired molecules by means of metabolic engineering and synthetic biology.

## Methods and Materials

### Strains and media

All the strains used in this study were listed in Table 1. The *Y*. *lipolytica* ATCC201249 was purchased from the American Type Culture Collection. *Y*. *lipolytica* strains were cultured at 28°C in YP medium [27] supplemented with the appropriate carbon source such as glucose, glycerol or sunflower seed oil. The engineered *Y*. *lipolytica* strains were selected on synthetic complete (SC) medium [11], which consists of 0.67% (w/v) yeast nitrogen base, 2% (w/v) glucose and the appropriate amino acid drop out mix supplemented. *E*. *coli* DH5α, which was used for vector construction and replication, was grown at 37°C in Luria-Bertani (LB) broth. If needed, 100 μg/ml ampicillin was added to the media prior to use.

**Table 1:** Strains used in this study.

Strain/Plasmid	Genotype or plasmid	Reference
**Plasmids**		
*pEASY*-Blunt	Blunt Cloning vector, resistant to aparamycin	TransGene
pUC57-Simple	Blunt Cloning vector, resistant to aparamycin	GenScript
*pEASY*-blunt-101	The cassette *ERG*5(700)-*URA*3-t0 was inserted into the NotI site of *pEASY*-blunt.	This study
*pEASY*-blunt-102	The cassette t1-*ERG*5(670) was inserted into the NotI site of *pEASY*-blunt.	This study
pUC57-Simple-101	pUC57-Simple harboring the cassette t0-EXP1p-XPR2t-t1	This study
pUC57-Simple-201	Gene *DHCR7* from *Xenapus laevis* was codon optimized, synthesized and cloned into pUC57-Simple-101	This study
pUC57-Simple-202	Gene *DHCR7* from *Oryza saliva* was codon optimized, synthesized and cloned into pUC57-Simple-101.	This study
pUC57-Simple-203	Gene *DHCR7* from *Rallus norvegicus* was codon optimized, synthesized and cloned into pUC57-Simple-101.	This study
pUC57-Simple-204	D409E mutation within *DHCR7* encoding sequences from pUC57-Simple-201	This study
***Yarrowia lipolytica* Strains**		
ATCC 201249	MATA, *ura3-302*, *leu2-270*, *lys8-11*, *pex17-ha*	[14]
SyBE_Yl1070028	*ERG5*::*URA3*-EXP1p-*DHCR7 (Xenapus laevis)*- XPR2t	This study
SyBE_Yl1070029	*ERG5*::*URA3*-EXP1p-*DHCR7 (Oryza saliva)*- XPR2t	This study
SyBE_Yl1070030	*ERG5*::*URA3*-EXP1p-*DHCR7 (Rallus norvegicus*, wild-type)-XPR2t	This study
SyBE_Yl1070031	*ERG5*::*URA3*-EXP1p-*DHCR7 (Xenapus laevis*, D409E)-XPR2t	This study

### Construction of campesterol producing strain

**Fig 1:**
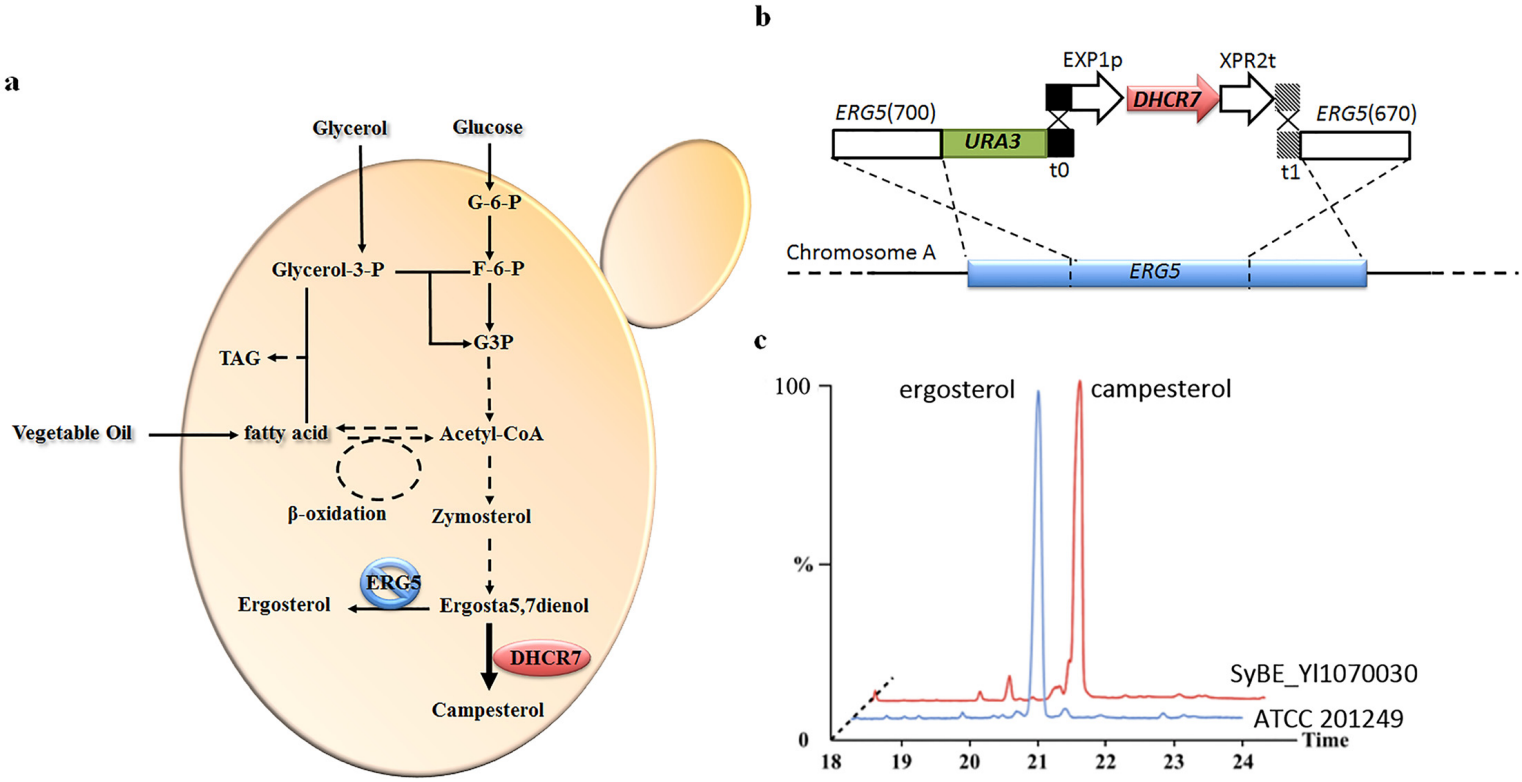
Overview of campesterol biosynthesis pathway and the corresponding genetic modification in *Y*. *lipolytica*. (**a**) Overview of campesterol biosynthesis pathway in *Y*. *lipolytica*. Campesterol was produced *via* expression of *DHCR7* and disruption of *ERG5* based on the ergosterol synthesis pathway in *Y*. *lipolytica*. Acetyl-CoA, the precursor of campesterol, is provided either by glycolysis from hydrophilic carbon sources (*i*.*e*. glucose and glycerol), or by β-oxidization from hydrophobic materials (*e*.*g*. oil). Meanwhile, acetyl-CoA from either hydrophilic or hydrophobic substrates can also generate cellular fatty acids and subsequently triacylglycerols (TAG), consisting of the *de novo* and the *ex novo* lipid accumulation process in *Y*. *lipolytica* respectively. (**b**) The method for expression of *DHCR7* and disruption of *ERG5* at the same time. The middle section of gene *ERG5* was replaced by the DHCR7 expression cassette (*URA3*-EXP1p-*DHCR7*-XPR2t) in chromosome A. (**c**) GC-TOF/MS profile of the wild-type *Y*. *lipolytica*. ATCC 201249 and campesterol producing strain SyBE_Yl1070030. Wild-type strain (blue) showed an ergosterol peak at 21 min, while *Y*. *lipolytica* SyBE_Yl1070028 (red) showed a campesterol peak at 21.7 min.

All the primers used in this study were listed in S1 Table. The front and the back section of gene *ERG5* (*ERG*5(700) and *ERG5*(670), Fig 1b), together with the *URA3* marker (orotidine 5'-phosphate decarboxylase encoding gene from *Y*. *lipolytica* for uracil synthesis) were amplified from the genomic DNA of *Y*. *lipolytica* with primer pairs of NotI-*ERG5*(700)-F/*URA3*-*ERG5*(700)-R, t1-*ERG5*(670)-F/t0-R and *ERG5*(700)-*URA*3-F/ t0-*URA*3-R, respectively. Meanwhile, the homologous region t0 and t1 (Fig 1b), which were the partial sequences of *Saccharomyces cerevisiae* terminator ENO2 and GPM1, were amplified from the genomic DNA of *S*. *cerevisiae* BY4742 with primer pairs of *URA*3-t0-F/NotI-t0-R and NotI-t1-F/*ERG5*(670)-t1-R. The cassette *ERG5*(700)-*URA3*-t0 and t1-*ERG5*(670) were assembled by overlap extension PCR (OE-PCR), then digested with NotI and introduced into the corresponding site of p*EASY*-Blunt (TransGene Biotech Co., China), obtaining p*EASY*-Blunt-101 and p*EASY*-Blunt-102.

The cassette t0-EXP1p-XPR2t-t1, which contained the endogenous promoter EXP1, terminator XPR2 [12, 28, 29] and two BsaI sites with opposite direction in between (S1 Fig), was synthesized by AuGCT Inc. (China) and cloned into pUC57-Simple (AuGCT Inc.). The genes *DHCR7* from *Rallus norvegicus*, *Oryza saliva* and *X*. *laevis* were codon optimized according to the codon bias of *Y*. *lipolytica* derived from the Codon Usage Database (http://www.kazusa.or.jp/codon/) (S2 Fig) and synthesized by GENEWIZ Inc. (China). The products were digested with BsaI and inserted into the corresponding site of the cassette t0-EXP1p-XPR2t-t1, producing DHCR7 expression cassette plasmids (S1 Fig), *i*.*e*. pUC57-Simple-201, pUC57-Simple-202 and pUC57-Simple-203 respectively. Mutagenesis (D409E) of DHCR7 encoding sequences from pUC57-Simple-201was achieved *via* the site-directed mutagenesis method described before [30], producing pUC57-Simple-204.

Four separate pUC57-Simple-DHCR7 expression cassettes (pUC57-Simple-201, pUC57-Simple-202, pUC57-Simple-203 and pUC57-Simple-204), together with *pEASY*-Blunt-101 and *pEASY*-Blunt-102 were digested with NotI, and co-transformed into the wild-type *Y*. *lipolytica* ATCC 201249 [31, 32]. The clone with mutant *ERG5*, whose middle section was replaced by DHCR7 expression cassette (URA3-EXP1p-DHCR7-XPR2t, Fig 1b), was selected on solid SC media without supplementation of uracil and further verified *via* PCR with primers of Y.L-*DHCR7*(rat440)-F/Y.L-*ERG5*cassette-R, Y.L-*DHCR7*(rice743)-F/Y.L-*ERG5*cassette -R and Y.L-*DHCR7* (toad459)-F/ Y.L-*ERG5*cassette-R respectively.

### Shake flask and fed-batch cultivation for campesterol production

For shake flask culture, engineered *Y*. *lipolytica* from glycerol tube was first grown in 5 ml SC medium without uracil at 28°C, 220 rpm for 24 h to the exponential phase (OD_600_≈5.0) and then inoculated into 30 ml corresponding SC medium at an initial OD_600_ of 0.2 for further cultivation of 18 h cultivation (OD_600_≈6.0). After that, the seed culture was transferred into 50 ml fresh YP medium supplemented with appropriate carbon source at an initial OD_600_ of 0.1 and grown until harvest. The concentration of glucose, glycerol and sunflower seed oil in the media was 5% (w/v), 4.07% (v/v) and 3.7% (v/v) respectively. Both glycerol and sunflower seed oil concentration were standardized in moles of carbon equivalents relating to 50 g/L glucose according to Sestric *et al*. [27].

For 5-L stirred-tank bioreactor (BLBIO-5GJG-2, Shanghai, China) experiment, precultures were inoculated into 500 ml YPD medium (YP medium with 5% glucose) with an initial OD_600_ of 0.2 and cultivated for 18 h until OD_600_ reached about 14. After that, 350 ml seed culture was transferred into 2.65 L of YP medium supplemented with 2% (w/v) glucose or 3.7% (v/v) sunflower seed oil as the carbon source. When glucose was employed as the carbon source, 50% (w/v) glucose solution was fed periodically into the fermentation medium to keep the glucose concentration under 1.0 g/L. Meanwhile, when sunflower seed oil was used as the carbon source, oil concentration was restricted below 2 g/L by adjusting feed rate after the batch oil was depleted. The temperature, pH, agitation and airflow were controlled at 28°C, 5.5, 400 rpm and 1 vvm, respectively. 15 ml cultures were collected every 12 h for the analysis of substrate concentration, cell density and campesterol production. When necessary, sterile water was added to the culture to maintain the volume of the fermentation at 3 L.

### Protein modeling

The amino acid sequences of DHCR7 from *R*. *norvegicus*, *O*. *saliva* and *X*. *laevis* were alignment by ClustalW2 (http://www.ebi.ac.uk/Tools/msa/clustalw2/). The DHCR7 models were developed with BioLuminate v1.8 Homology Modeling from Schrodinger. The crystal structure of delta14-sterol reductase from *M*. *alcaliphilum* 20Z (PDB accession 4quv) [20] was used as the template in all DHCR7 modeling. The sequence identity between DHCR7 proteins and the template ranged from 33% to 39%. As part of the NADPH was missing in the template, the Nicotinamide adenine group was re-modeled. The ergosta-5,7-dienol substrate was docked to DHCR7 using AutoDock Vina [33]. The mutation on *X*. *laevis* DHCR7 was carried out using *Residue Scanning* Tools included in the BioLuminate suite.

### Microscopic analysis and biomass determination

For microscopic observation, cells were harvested by centrifugation at 10,000 rpm for 1 min and re-suspended with sterile water to dilute into the final cell density of OD_600_ to 5.0. Images were taken with an Olympus CX41 (Olympus, Tokyo, Japan) at the magnification of 40×. Biomass determination was performed according to Aggelis *et al*. [26] and Papanikolaou *et al*. [16]. Especially when sunflower seed oil was used as the carbon source, the harvested cells were washed three times with hexane, followed by rinsing three times with distilled water in order to remove residual fat before the dry cell weight (DCW) measurement [16, 26].

### Measurement of substrate (glucose, glycerol, sunflower oil) concentration and cellular total lipid content

The glucose and glycerol concentration in the medium were analyzed with HPLC following the methods described before [34]. Unconsumed lipid were extracted and measured according to Papanikolaou *et al*. [16]. Cellular total lipid was extracted and measured according to Soxhlet extraction procedure revised from Folch e*t al* [35]. Five grams of dried cells were weighed in a thimble and subjected to Soxhlet extraction with 80 mL of diethyl ether for 2 hour at 60°C. During the extraction process, all the solvent was collected in a round-bottomed flask (the weight of the flask M1). After extraction and evaporation of solvent, the round-bottomed flask was further dried at 104°C for 30 min until the weight was constant (M2). The total lipid content (TL %) was calculated as follows,

$$\text{TL\%}=\frac{\left(\text{M}2-\text{M}1\right)}{\text{DCW}}\times 100\%$$

### Analysis of campesterol production

According to the earlier works [3, 4, 7, 34], saponification with KOH-methanol solution in grinded cells has been prove to be an efficient way to recover sterol from its ester fraction. Compared with the saponificated samples, ethyl esterified campesterol was detected beside the free form in the product without saponification (S3 Fig). Even though the esterified form only occupied a little fraction of total campesterol (S3 Fig), saponification is still required in sample treatment before GC-TOF/MS analysis.

To be specific, cells were harvested, and washed by Milli-Q double deionized water. After centrifugation, cell pellet was grounded into fine powder in liquid nitrogen, and then transferred into a 10 ml centrifugal tube, suspended in 5 ml KOH-methanol solution (20% (w/v) KOH, 80% (v/v) methanol) and heated for 5 h at 60°C for saponification. 2 ml hexane was added into the tube to extract campesterol and then freeze dried overnight. Then the product was resuspended with 400 μl n-hexane and refreeze dried overnight. The derivatization of the freeze-dried product was conducted with 200 μl N-methyl-N-(trimethylsilyl) trifluoroacetamide (MSTFA) at 30°C for 2 h. Samples were diluted with hexane and measured by GC-TOF/MS (Waters Corp. USA) [34]. The injector temperature was 280°C. The column temperature was initially maintained at 70°C for 1 min, then increased to 250°C at a temperature ramp of 20°C/min and maintained at 250°C for 2 min, followed by an increase to 280°C at a temperature ramp of 15°C/min, finally maintained at 280°C for 15 min. The campesterol was identified by Nist library search 2006 (the mass fragment 472, 382, 343 and 129 m/z).

## Results and Discussion

### Campesterol synthesis pathway was constructed in *Y*. *lipolytica via* blocking ergosterol biosynthesis and introducing heterogeneous DHCR7

The endogenous ergosterol synthesis pathway is the competitive branch to the heterogeneous production of campesterol (Fig 1a). To produce campesterol in *Y*. *lipolytica*, the middle section of the gene *ERG5* was replaced by the heterologous DHCR7 expression cassette. The expression of *DHCR7* was controlled by the EXP1 promoter of *Y*. *lipolytica* (Fig 1b). The strain contained *R*. *norvegicus* DHCR7 (named as SyBE_Yl1070030) was cultivated in YPD medium in shake flask for 140 h and the product was analyzed by GC-TOF/MS. Instead of ergosterol (at 21 min) in the wild-type *Y*. *lipolytica* ATCC201249, campesterol (at 21.7 min) was the major product in strain SyBE_Yl1070030 (Fig 1c). Moreover, there was no other distinct by-product accumulation (Fig 1c), indicating the campesterol biosynthesis pathway was successfully functioned in *Y*. *lipolytica*.

### Strain with DHCR7 from *X*. *laevis* achieved the highest campesterol production

**Fig 2:**
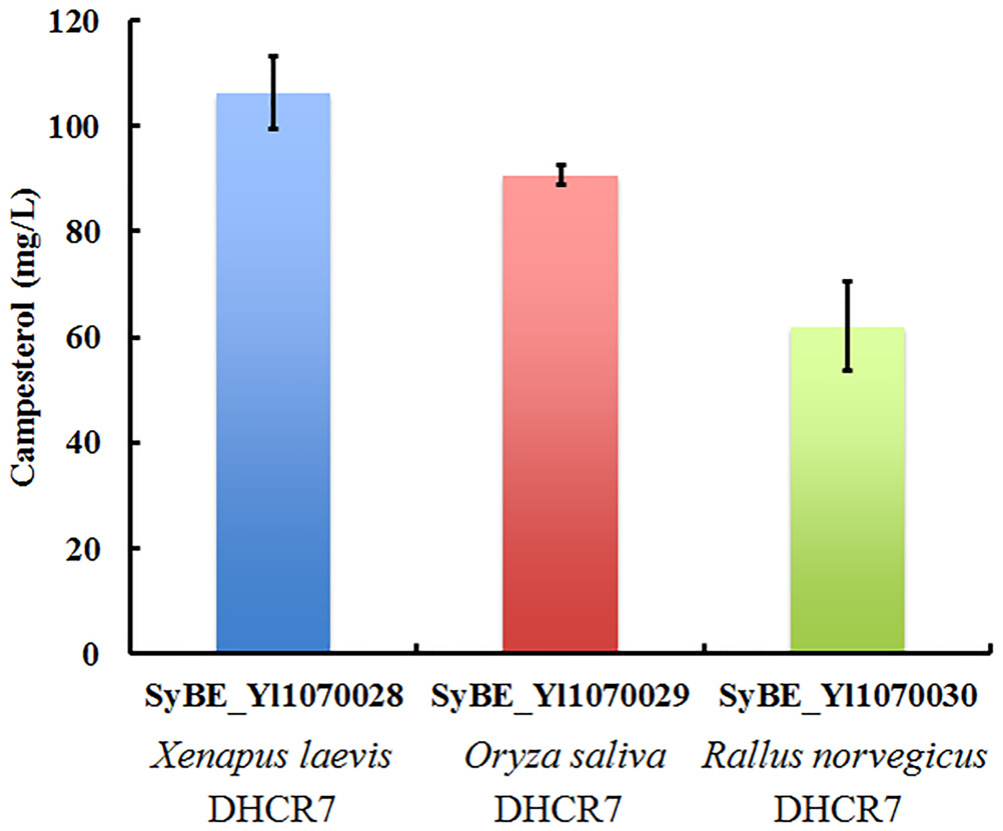
Campesterol production in strains with DHCR7 from different species. *Y*. *lipolytica* strains with DHCR7 from *X*. *laevis*, *O*. *saliva* and *R*. *norvegicus* were named as SyBE_Yl1070028, SyBE_Yl1070029 and SyBE_Yl1070030 respectively. The amount of campesterol produced by these three strains was measured after 120 h shake flask culture in YPD medium.

The gene *DHCR7* was the only heterogeneous gene and essential in campesterol synthesis pathway. Besides the one from *R*. *norvegicus*, DHCR7 from other two species as *O*. *saliva* and *X*. *laevis* were selected to construct campesterol producing strain, obtaining strains SyBE_Yl1070029 and SyBE_Yl1070028. Together with strain SyBE_Yl1070030, all these three strains were cultured in YPD medium in shake flask for campesterol production. The strain SyBE_Yl1070028 with DHCR7 from *X*. *laevis* achieved the highest titer of 106±8.5 mg/L (Fig 2). Thus, this strain was used as the candidate for the further optimization on campesterol production.

**Fig 3:**
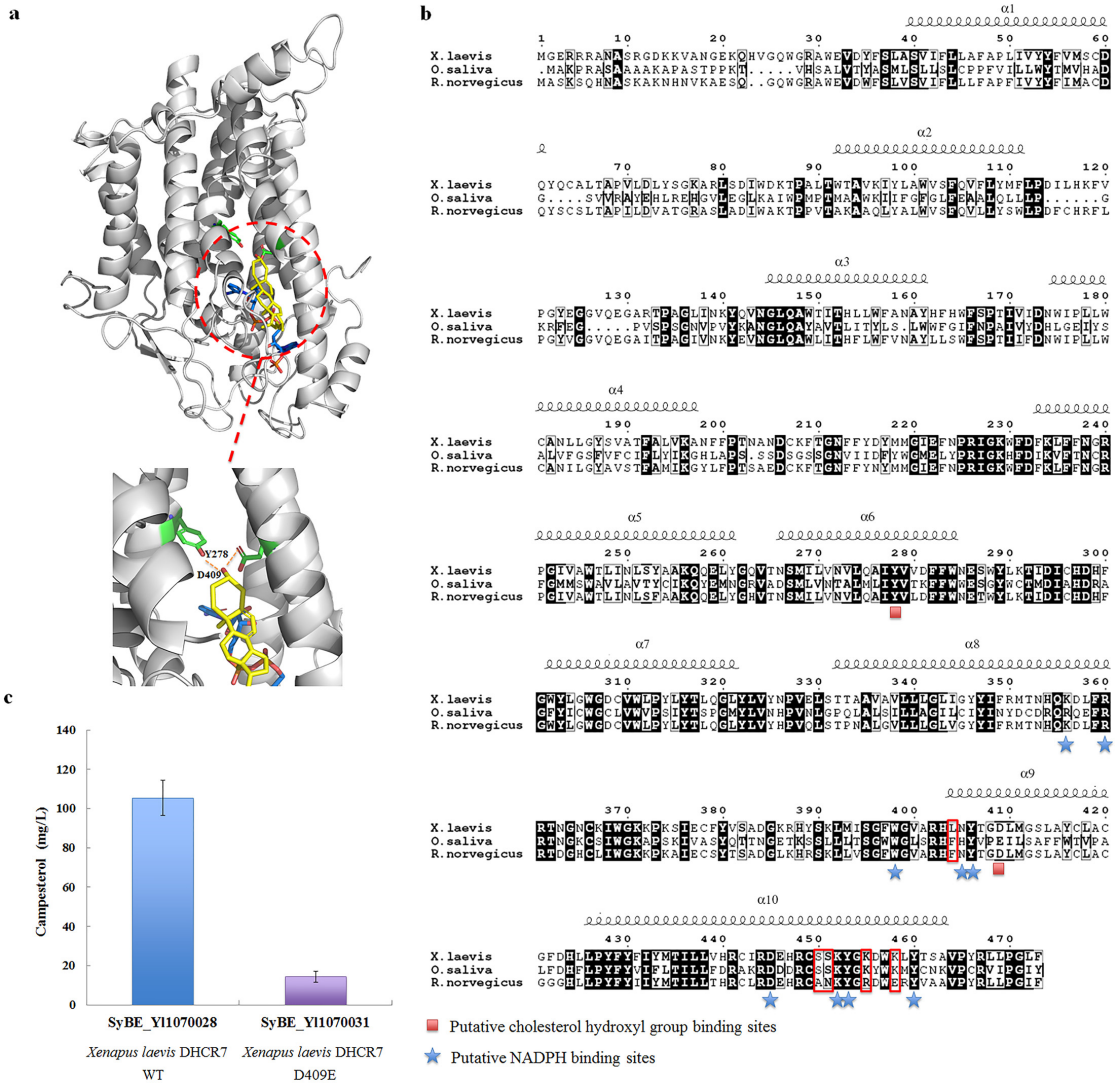
Sequences and architecture differences among DHCR7 from *X*. *laevis*, *O*. *saliva* and *R*. *norvegicus*. (**a**) Overview the structure model of DHCR7 from *X*. *laevis*. NADPH (blue) was docked into the NADPH pocket and ergosta-5,7-dienol (yellow) modelled into the substrate binding pocket in the DHCR7 structure. The pocket for substrate binding was enlarged and present below, indicating that Y278 and D409 clamp the hydroxyl of the substrate through a hydrogen-bonding network. (**b**) Sequences alignment of DHCR7 from *X*. *laevis*, *O*. *saliva and R*. *norvegicus*. The putative cholesterol hydroxyl group binding sites and cofactor NADPH binding sites were marked by red squares and blue stars, respectively. Mutations which might attribute to the lowest activity of DHCR7 from *R*. *norvegicus* were boxed with red lines. (**c**) Campesterol production in *Y*. *lipolytica* strains with wild-type and mutated *X*. *laevis* DHCR7 (named as SyBE_Yl1070028 and SyBE_Yl1070031 respectively). The campesterol production for these strains was measured after 120 h shake flask culture in YPD medium.

Based on the crystal structure of its homological protein delta14-sterol reductase (MaSR1) from *M*. *alcaliphilum* 20Z (PDB accession 4quv), the structural model of the full-length DHCR7 from *X*. *laevis*, *O*. *saliva* and *R*. *norvegicus* was generated. As shown in Fig 3a, DHCR7 contained ten helices, nine of them (1–9 helix) were reported to be transmembrane segments [19]. The 6–10 helixes enveloped two interconnected pockets for NADPH and sterol binding, respectively (Fig 3a). Notably in the putative sterol-binding pocket of *X*. *laevis* DHCR7, the hydroxyl group on the substrate formed a favorable hydrogen-bonding network with Y278 and D409 (Fig 3a), which led to a tight binding of substrate to the enzyme. In DHCR7 from *O*. *saliva*, this particular Asp residue was mutated to Glu (Fig 3b). And *in silico* computation calculation using this model showed a loss of ~4 kcal/mol in binding affinity caused by the mutation D409E. For validation, when D409 within *X*. *laevis* DHCR7 of SyBE_Yl1070028 was mutated into Glu, the production of campesterol decreased by 86.7% accordingly (Fig 3c), suggesting that D409 was essential for substrate binding and the mutation D409E might be the reason for lower campesterol production achieved from strain SyBE_Yl1070029 (possessing *O*. *saliva* DHCR7) than strain SyBE_Yl1070028 as well.

However, according to the current model, it was hard to explain the difference in campesterol production between the strain possessing DHCR7 from *X*. *laevis* and *R*. *norvegicus*, since DHCR7 from these two species had identical active sites (Fig 3b). Through sequence alignment, some mutations such as L404F, S450A, S451N, K455R and K460E were identified (Fig 3b), which might attribute to the lowest activity of DHCR7 from *R*. *norvegicus*. These sites still need to be verified *via* point mutagenesis and activity assay in future study.

### Sunflower seed oil as carbon source achieved the highest reported campersterol titer

**Fig 4:**
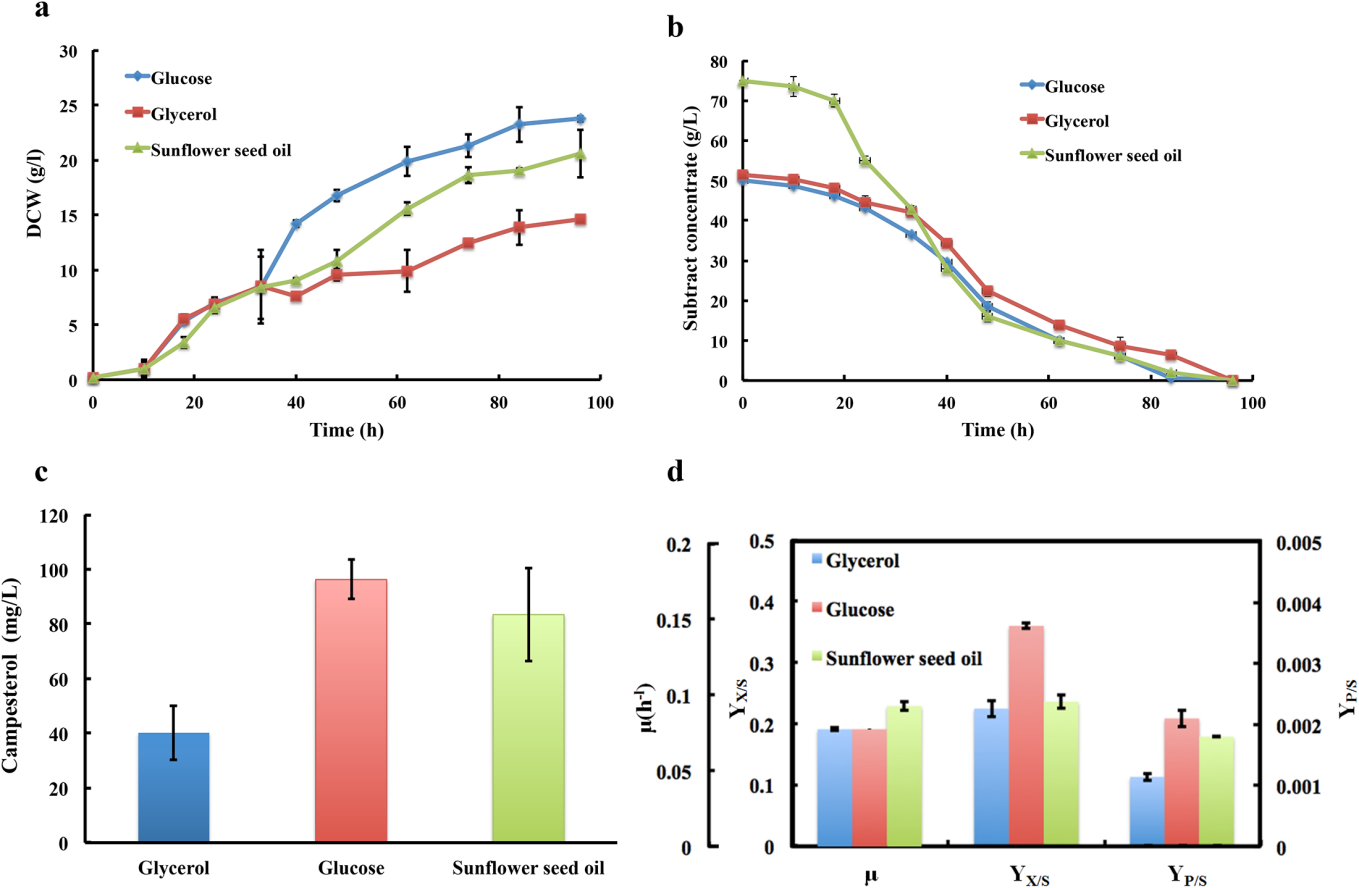
Shake flask cultivation with different carbon sources. (**a**) Cells growth for *Y*. *lipolytica* strain SyBE_Yl1070028 with glucose, glycerol and sunflower seed oil as the carbon source. (**b**) Substrate (glucose, glycerol and sunflower oil) consumed by strain SyBE_Yl1070028. (**c**) The final campersterol titers produced by strain SyBE_Yl1070028 with glucose, glycerol and sunflower seed oil as the carbon source. (**d**) Cells growths specific rate (μ, h^-1^), biomass conversion yield (Y_X/S_) and campesterol production yield (Y_P/S_) for strain SyBE_Yl1070028 with different carbon source. μ is determined as ln(X/X_0_)/ (t-t_0_), where X is for total biomass (g/L) and t is for time (h). Meanwhile, Y_X/S_ represents the yield of total biomass produced per unit of substrate consumed and Y_P/S_ represents the yield of product formed per unit of substrate consumed.

The effect of different carbon source was investigated to optimize fermentation process. Glucose, glycerol and sunflower seed oil were chosen as the carbon source to diverse production of campesterol in shake flasks. And the biomass produced and the substrate consumed in time course by *Y*. *lipolytica* strain SyBE_Yl1070028 together with the final campersterol titers were shown in Fig 4a, 4b and 4c, respectively. As shown in Fig 4d, no matter the cell specific growth rate, the biomass conversion yield and the campesterol production yield, glycerol was not a good carbon source for campesterol production. In terms of cell growth, the engineered *Y*. *lipolytica* strain with sunflower seed oil displayed a bit higher cell growth rate than that with glucose (Fig 4d). Nevertheless, higher biomass conversion yield and a bit higher campesterol yield were achieved in presence of glucose (Fig 4d). Thus except glycerol, both glucose and sunflower seed oil were selected as the potential carbon source for further process optimization in bioreactor.

**Fig 5:**
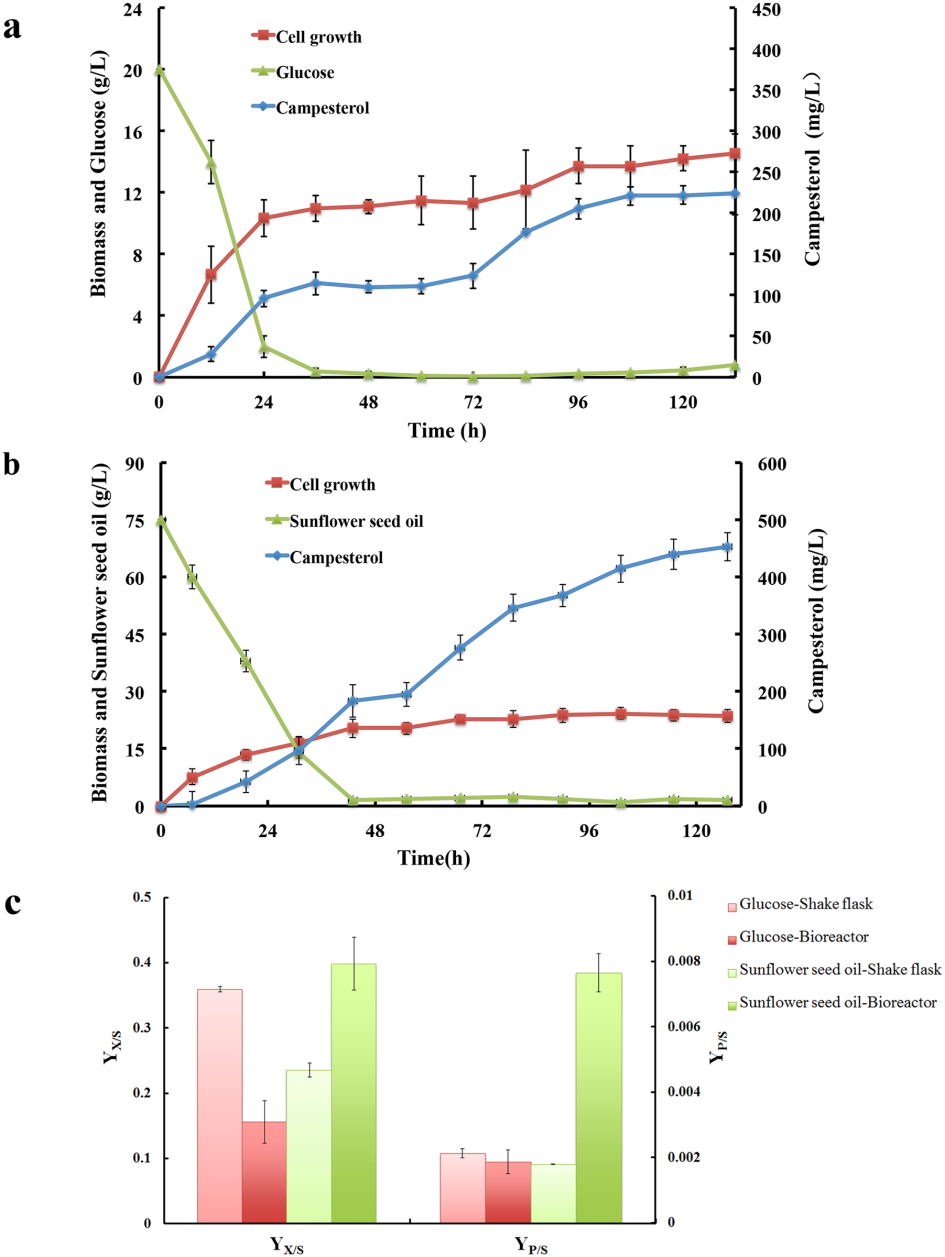
Bioreactor fermentation with different carbon sources. Campesterol producing strain SyBE_Yl1070028 was fed-batch cultured in glucose (**a**) or in sunflower seed oil (**b**) with carbon source restriction strategy. Y_X/S_ (yield of total biomass produced per unit of substrate consumed) and Y_P/S_ (yield of product formed per unit of substrate consumed) for fed-batch bioreactor cultures were compared with those from the shake-flask experiments (**c**).

All the fermentation optimization experiments were conducted in 5-L stirred bioreactor. The rationale of experiment design is aimed to achieve high cell density fermentation by carbon source restriction strategy. In case of glucose as the carbon source, after the initial glucose was depleted in batch medium, glucose solution was fed periodically into the medium. The glucose concentration was maintained lower than 1 g/L by controlling the feeding rate (Fig 5a). No acetate was observed during the whole process. Eventually, a titer of 224±25.9 mg/L campesterol was obtained after 132 h cultivation (Fig 5a). However, compared to those in shake flask, the biomass conversion yield and the campesterol yield reduced by 23.8% and 12.7% respectively (Fig 5c), probably due to lacking other nutritions such as nitrogen source and metal ions during this period. These would be a potential optimization direction for future. In the meanwhile, the fermentation was also carried out using sunflower seed oil as the carbon source. To realize carbon restriction, the sunflower seed oil concentration was restricted below 2 g/L by adjusting the feed rate after oil was almost used up in batch medium at 43 h (Fig 5b). There was no acetate accumulated in this case as well. Consequently, a highest reported microbial titer known of 453±24.7 mg/L campesterol was generated (Fig 5b). In particularly, the biomass conversion yield and the product yield were 39.9% and 0.765% respectively, which were significantly increased by 69.2% and 328% compared to those in shake flask (Fig 5c). Thus, oil would be a candidate carbon source for campesterol production in engineered *Y*. *lipolytica*.

**Fig 6:**
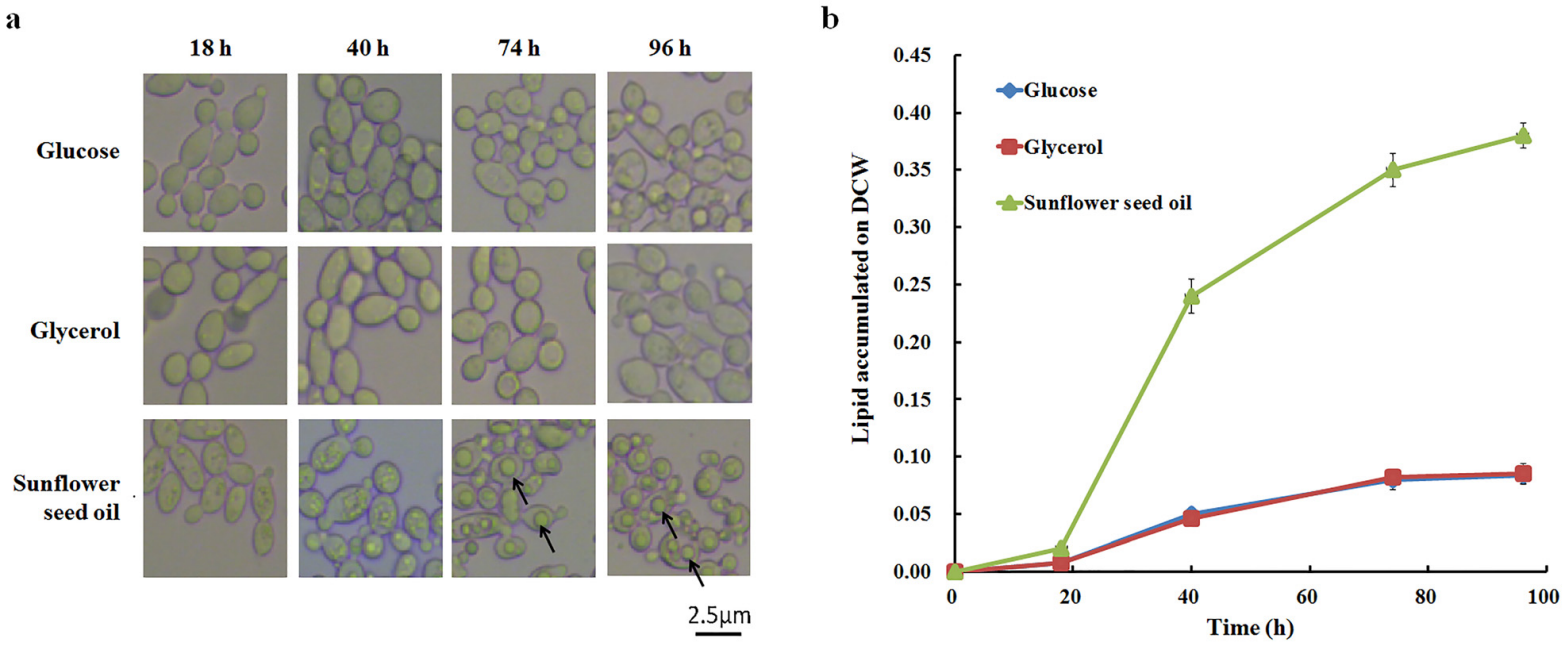
Lipid accumulation in *Y*. *lipolytica* strain SyBE_Yl1070028 cultured with different carbon sources. (**a**) The morphology and the lipid body (indicated by arrows) of the engineered *Y*. *lipolytica* strain in shake flask cultivation with glucose, glycerol and sunflower seed oil as the carbon source. (**b**) The lipid accumulation on the dry cell weight (DCW) basis for the shake flask trials.

*Y*. *lipolytica* lipid bodies are synthesized either through *de novo* lipid accumulation from hydrophilic carbon sources (*e*.*g*. glucose and glycerol, Fig 1a) or through *ex novo* lipid accumulation from hydrophobic materials (*e*.*g*. oil, Fig 1a). As the main component of oil, fatty acids could enter β-oxidation and be degraded into acetyl-coA [36–39], which is an important precursor for both campesterol and lipids synthesis (Fig 1a). Besides that, fatty acids could also be converted to triglycerides and stored as lipid bodies [39]. It is reported that the size of lipid bodies is influenced by different carbon sources [15, 40]. And in this work, the lipid bodies were only observed distinctively when providing sunflower seed oil as the carbon source and the size was expanding over time course (Fig 6a), which was consistent with the early works [15, 16, 26]. Consequently, 0.38 g of lipids per g of dry matter was observed when sunflower seed oil was employed as the sole carbon source (Fig 6b).

Because *Y*. *lipolytica* lipid bodies has been proved to be an advantageous cellular component to store less polar compounds [6], the microbial lipids accumulation seems be benefit to campesterol production by providing a storage space. However, unlimited expansion on the size of lipid bodies would not end up to enhancing campesterol production. In fact, there are two kinds of complex flux flow competition between campesterol production and lipid storage, *i*.*e*. one in fatty acids flux flow towards between acetyl-coA synthesis and lipid bodies’ formation, and another one in acetyl-coA flux flow to sterol production and back to lipids synthesis (Fig 1a). Thus, how to balance these two aspects of flux would be a key point to enhance campesterol titer. In this study, under current carbon restriction strategy, the campesterol production yield increased by 328% compared to that in shake flask. However, this is still not considered as a high yield, and more efforts would be taken in further study for developing optimal fermentation process to better balance the formation rate between lipid bodies and targeted product.

## Conclusion

*Y*. *lipolytica* was engineered to produce campesterol in this study. The DHCR7 from *X*. *laevis* was verified to be the best one for campesterol among those from *R*. *norvegicus*, *O*. *saliva* and *X*. *laevis*. Moreover, the highest campesterol production titer was achieved in 5-L bioreactor using sunflower seed oil as the sole carbon source, suggesting oil would be a promising carbon source for campesterol production in *Y*. *lipolytica*. This study sets a good reference for the heterologous biosynthesis enhancement of desired products by combination of enzyme and carbon source screening.

## Supporting Information

S1 Fig Construction of *DHCR7* expression cassette plasmid. (DOCX)

S2 Fig The Codon-optimized sequence of *DHCR7* from *R*. *norvegicus*, *O*. *saliva* and *X*. *laevis*. (DOCX)

S3 Fig GC-TOF-MS analysis of *Y*. *lipolytica* strain SyBE_Yl1070028. (DOCX)

S1 Table Primers used in this study. (DOCX)
