# Supplementary figures and images for: Engineering Yarrowia lipolytica for Campesterol Overproduction
Source: PLoS One. 2016 Jan 11;11(1):e0146773. doi: 10.1371/journal.pone.0146773 (PMC4709189; doi:10.1371/journal.pone.0146773)

**S1 Fig. Construction of *DHCR7* expression cassette plasmid**

**
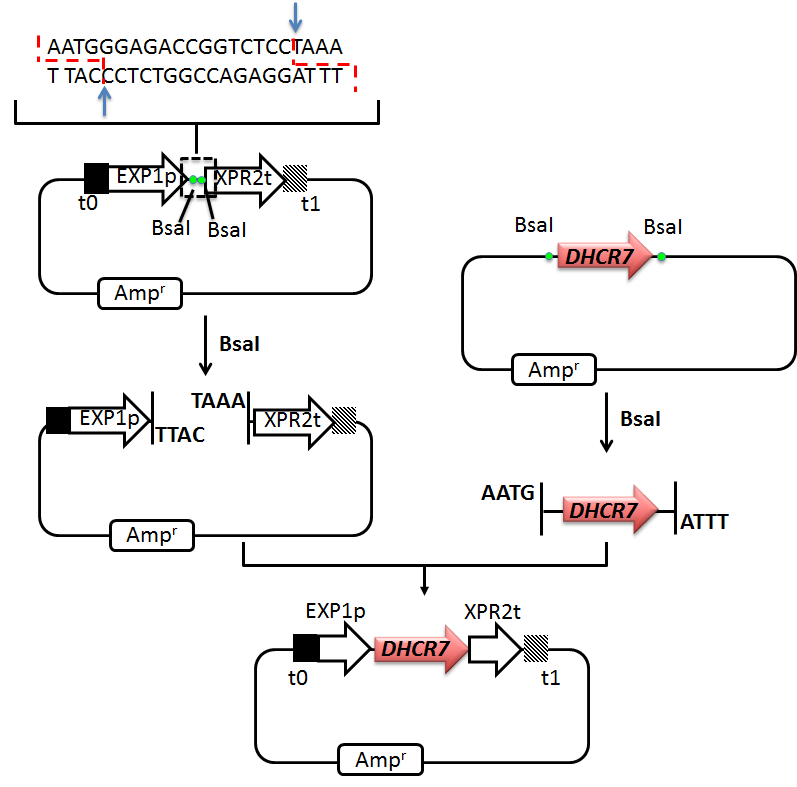
**

Supplement: S1 Fig — (DOCX) [file pone.0146773.s001.docx]
