# Supplementary material for: Engineering Yarrowia lipolytica for Campesterol Overproduction
Source: PLoS One. 2016 Jan 11;11(1):e0146773. doi: 10.1371/journal.pone.0146773 (PMC4709189; doi:10.1371/journal.pone.0146773)
Supplement: S2 Fig — (DOCX) [file pone.0146773.s002.docx]

**S2 Fig. The Codon-optimized sequences of *DHCR7* from *R. norvegicus*, *O. saliva* and *X. laevis***

***a. DHCR7* from *R. norvegicus：***

GCGGCCGCGGTCTCCCAAAATGGCTTCTAAGTCTCAGCACAACGCTTCTAAGGCTAAGAACCACAACGTTAAGGCTGAGTCTCAGGGTCAGTGGGGTCGAGCTTGGGAGGTTGACTGGTTCTCTCTGGTTTCTGTTATCTTCCTGCTGCTGTTCGCTCCCTTCATCGTTTACTACTTCATCATGGCTTGTGACCAGTACTCTTGTTCTCTGACCGCTCCCATCCTGGACGTTGCTACCGGTCGAGCTTCTCTGGCTGACATCTGGGCTAAGACCCCCCCCGTTACCGCTAAGGCTGCTCAGCTGTACGCTCTGTGGGTTTCTTTCCAGGTTCTGCTGTACTCTTGGCTGCCCGACTTCTGTCACCGATTCCTGCCCGGTTACGTTGGTGGTGTTCAGGAGGGTGCTATCACCCCCGCTGGTATCGTTAACAAGTACGAGGTTAACGGTCTGCAGGCTTGGCTGATCACCCACTTCCTGTGGTTCGTTAACGCTTACCTGCTGTCTTGGTTCTCTCCCACCATCATCTTCGACAACTGGATCCCCCTGCTGTGGTGTGCTAACATCCTGGGTTACGCTGTTTCTACCTTCGCTATGATCAAGGGTTACCTGTTCCCCACCTCTGCTGAGGACTGTAAGTTCACCGGTAACTTCTTCTACAACTACATGATGGGTATCGAGTTCAACCCCCGAATCGGTAAGTGGTTCGACTTCAAGCTGTTCTTCAACGGTCGACCCGGTATCGTTGCTTGGACCCTGATCAACCTGTCTTTCGCTGCTAAGCAGCAGGAGCTGTACGGTCACGTTACCAACTCTATGATCCTGGTTAACGTTCTGCAGGCTATCTACGTTCTGGACTTCTTCTGGAACGAGACGTGGTACCTGAAGACCATCGACATCTGTCACGACCACTTCGGTTGGTACCTGGGTTGGGGTGACTGTGTTTGGCTGCCCTACCTGTACACCCTGCAGGGTCTGTACCTGGTTTACCACCCCGTTCAGCTGTCTACCCCCAACGCTCTGGGTGTTCTGCTGCTGGGTCTGGTTGGTTACTACATCTTCCGAATGACCAACCACCAGAAGGACCTGTTCCGACGAACCGACGGTCACTGTCTGATCTGGGGTAAGAAGCCCAAGGCTATCGAGTGTTCTTACACCTCTGCTGACGGTCTGAAGCACCGATCTAAGCTGCTGGTTTCTGGTTTCTGGGGTGTTGCTCGACACTTCAACTACACCGGTGACCTGATGGGTTCTCTGGCTTACTGTCTGGCTTGTGGTGGTGGTCACCTGCTGCCCTACTTCTACATCATCTACATGACCATCCTGCTGACCCACCGATGTCTGCGAGACGAGCACCGATGTGCTAACAAGTACGGTCGAGACTGGGAGCGATACGTTGCTGCTGTTCCCTACCGACTGCTGCCCGGTATCTTCTAAATTGGGAGACCGCGGCCGC

***b. DHCR7* from *O. saliva:***

GCGGCCGCGGTCTCCAATGGCTAAGCCCAGAGCTTCCGCTGCTGCCGCTAAGGCCCCCGCTTCTACTCCCCCTAAGACCGTGCACTCCGCCCTGGTCACCTACGCTTCCATGCTGTCCCTGCTGTCCCTCTGCCCTCCCTTCGTGATCCTGCTCTGGTATACCATGGTGCACGCCGACGGCTCTGTGGTCAGAGCTTACGAGCACCTCCGAGAGCACGGCGTCCTCGAGGGCCTCAAGGCCATTTGGCCCATGCCTACCATGGCCGCCTGGAAGATCATCTTTGGTTTCGGTCTGTTCGAGGCCGCCCTCCAGCTCCTGCTGCCCGGTAAGCGATTCGAGGGCCCTGTGTCCCCTTCCGGTAACGTCCCCGTCTATAAGGCTAACGGCCTGCAAGCCTACGCTGTGACCCTGATCACCTACCTCTCCCTGTGGTGGTTCGGCATCTTTAACCCCGCCATCGTGTACGACCACCTGGGTGAGATCTACTCTGCCCTGGTCTTCGGTTCCTTCGTGTTCTGCATCTTCCTCTACATTAAGGGCCACCTCGCCCCCTCTTCCTCCGACTCCGGTTCCTCCGGCAATGTGATCATCGACTTCTACTGGGGTATGGAGCTCTACCCTCGAATCGGCAAGCACTTCGACATCAAGGTCTTTACCAACTGTAGATTCGGCATGATGTCTTGGGCCGTGCTGGCCGTCACCTACTGCATTAAGCAGTACGAAATGAACGGCCGAGTCGCCGACTCTATGCTCGTCAACACCGCCCTGATGCTGATCTACGTCACCAAGTTCTTTTGGTGGGAGTCCGGCTACTGGTGCACTATGGACATCGCTCATGACAGAGCCGGCTTCTACATTTGCTGGGGCTGCCTGGTCTGGGTCCCTTCCATTTACACCTCCCCTGGCATGTATCTCGTGAACCACCCCGTGAACCTCGGTCCCCAGCTGGCTCTCTCCATTCTGCTGGCTGGCATCCTGTGCATTTACATCAACTACGATTGTGACCGACAGCGACAGGAGTTCCGACGAACCAACGGCAAGTGTTCCATTTGGGGTAAGGCCCCCTCTAAGATCGTGGCTTCCTACCAGACCACCAACGGTGAGACGAAGTCCTCCCTCCTCCTGACCTCTGGCTGGTGGGGTCTGTCCCGACATTTCCACTACGTGCCCGAGATCCTGTCCGCCTTTTTCTGGACCGTGCCCGCCCTCTTCGATCACTTCCTGCCTTACTTCTACGTCATCTTCCTCACCATCCTCCTCTTCGACCGAGCCAAGAGAGATGACGACCGATGCTCCTCCAAATACGGCAAGTATTGGAAAATGTACTGCAACAAGGTCCCTTGCCGAGTGATCCCCGGTATTTACTAAAGGAGACCGCGGCCGC

***c. DHCR7* from *X. laevis:***

GCGGCCGCGGTCTCCAATGGGTGAGCGTCGCCGCGCAAATGCCAGCCGTGGTGATAAGAAGGTGGCCAATGGCGAGAAGCAGCATGTTGGCCAGTGGGGTCGCGCATGGGAAGTGGATTACTTTAGCCTGGCCAGCGTGATTTTTCTGCTGGCCTTCGCCCCGCTGATTGTTTATTATTTCGTGATGAGCTGCGATCAGTACCAGTGCGCCCTGACCGCACCGGTTCTGGACCTGTATAGCGGTAAAGCCCGCCTGAGCGACATTTGGGATAAAACCCCGGCCCTGACCTGGACCGCAGTGAAAATCTACCTGGCCTGGGTGAGCTTCCAAGTGTTTCTGTACATGTTCCTGCCGGACATCCTGCACAAATTCGTGCCTGGCTACGAAGGTGGTGTTCAAGAGGGGGCACGCACCCCTGCCGGTCTGATCAACAAGTACCAGGTGAATGGCCTGCAGGCCTGGACCATCACACATCTGCTGTGGTTTGCCAACGCATACCACTTCCACTGGTTCAGCCCGACCATTGTTATCGACAACTGGATCCCGCTGCTGTGGTGTGCCAATTTACTGGGCTACAGCGTGGCAACCTTCGCACTGGTGAAAGCCAACTTCTTCCCGACAAACGCAAATGATTGTAAGTTCACCGGCAACTTTTTCTATGACTACATGATGGGCATCGAATTCAACCCGCGCATTGGCAAATGGTTCGATTTCAAGCTGTTCTTTAACGGTCGCCCGGGCATCGTGGCCTGGACCCTGATCAACCTGAGCTATGCCGCAAAACAGCAGGAGCTGTATGGCCAGGTTACCAACAGCATGATTCTGGTGAACGTGCTGCAGGCCATTTACGTGGTTGACTTCTTCTGGAATGAAAGCTGGTATCTGAAAACCATTGATATTTGCCACGATCATTTTGGCTGGTATCTGGGTTGGGGTGACTGCGTGTGGCTGCCGTACTTATACACACTGCAGGGCCTGTACCTGGTTTACAACCCGGTGGAACTGAGCACAACAGCCGCCGTGGCCGTTTTACTGCTGGGCCTGATCGGCTACTACATATTCCGTATGACCAATCATCAGAAAGACCTGTTTCGCCGCACCAATGGCAATTGCAAGATTTGGGGTAAAAAACCGAAGAGCATTGAGTGCTTCTACGTGAGCGCCGACGGCAAGCGCCATTACAGCAAACTGATGATTAGCGGCTTTTGGGGCGTGGCCCGTCATCTGAATTACACCGGCGATCTGATGGGCAGCCTGGCATATTGCCTGGCCTGCGGCTTTGACCATCTGCTGCCGTATTTTTATTTTATTTATATGACCATTCTGCTGGTGCATCGTTGCATTCGCGACGAACATCGCTGCAGCAGCAAATACGGCAAGGACTGGAAACTGTATACCAGCGCAGTGCCGTATCGCTTACTGCCGGGTCTGTTTTAAAGGAGACCGCGGCCGC
