# Supplementary material for: Engineering Yarrowia lipolytica for Campesterol Overproduction
Source: PLoS One. 2016 Jan 11;11(1):e0146773. doi: 10.1371/journal.pone.0146773 (PMC4709189; doi:10.1371/journal.pone.0146773)

**S3 Fig. GC-TOF-MS analysis of *Y. lipolytica* strain SyBE_Yl1070028.** Strain SyBE_Yl1070028 was shake-flask cultured in YPD medium for 120 h. All the harvested cells were grounded into fine powder in liquid nitrogen and distributed equally into three parts. One was treated with both saponification and derivatization (red), one was treated with derivatization but not saponification (green) and the last one was treated with neither derivatization nor saponification (purple). After pretreatment, all the samples were analyzed by GC-TOF-MS.


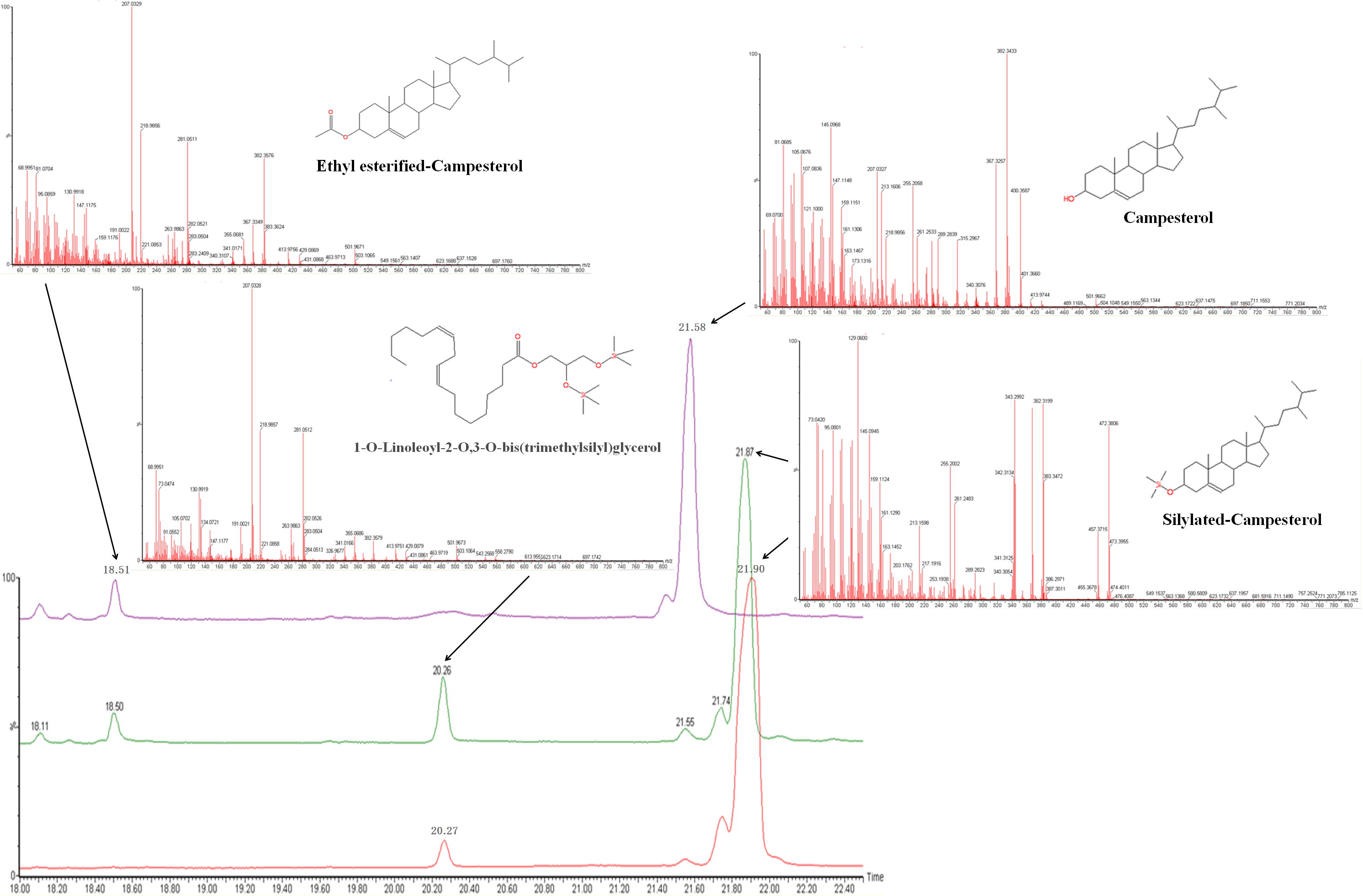

Supplement: S3 Fig — (DOCX) [file pone.0146773.s003.docx]
