# Supplementary material for: Engineering Yarrowia lipolytica for Campesterol Overproduction
Source: PLoS One. 2016 Jan 11;11(1):e0146773. doi: 10.1371/journal.pone.0146773 (PMC4709189; doi:10.1371/journal.pone.0146773)
Supplement: S1 Table — (DOCX) [file pone.0146773.s004.docx]

**S1 Table. Primers used in this study**

| Name | Sequence (5’-3’) |
| --- | --- |
| Not1-*ERG5*(700)-F | AAAAGCGGCCGCATGAACGCTACCCAACCGGAGT |
| *URA3*- *ERG5* (700)-R | GAGAATGCTCCACAGAACACACCTGAAGGGCAGAATGA |
| *ERG5* (700)-*URA3*-F | CCCCATCATTCTGCCCTTCAGGTGTGTTCTGTGGAGCATTC |
| t0-*URA3*-R | AAAGACTAATAATTCTTAGTTAAAAGCACTGGTGTAGTGGTAGTGCAGTGGT |
| *URA3*-t0-F | CACTGCACTACCACTACACCAGTGCTTTTAACTAAGAATTATTAGTCTT |
| Not1-t0-R | AAAAGCGGCCGCAGCCACGCGTGTGCACC |
| Not1-t1-F | GCGGCCGCGTCTGAAGAATGAATGATTTGATG |
| *ERG5* (670)-t1-R | CAAAGTCCCGGCCGTTCATGTAATAATACTCCTCAAATTGCTACCAC |
| t1- *ERG5* (670)-F | TAGCAATTTGAGGAGTATTATTACATGAACGGCCGGGAC |
| t0-R | AAAAGCGGCCGCAGCCACGCGTGTGCACC |
| Y.L-EXP1p-F | CCTTGTTTAGTTTGGCTCCTGCCCGTTTCG |
| Y.L-XPR2t-R | TCACAGAGGGAATTGATGAACAAAGACG |
| Y.L-*DHCR7* (rat440)-F | GGCTGATCACCCACTTCCT |
| Y.L-*DHCR7* (rice743)-F | CGAGTCGCCGACTCTATGC |
| Y.L-*DHCR7* (toad459)-F | CCTGGACCATCACACATCTG |
| Y.L-*ERG5*cassette -R | CAGAACTGCTGGCCCTACTC |
| DHCR7(D409E)-F | CTGATGGGCAGCCTGGC |
| DHCR7(D409E)-R | CTCGCCGGTGTAATTCAGATGAC |
